# Supplementary material for: Post-capillary venules are the key locus for transcytosis-mediated brain delivery of therapeutic nanoparticles
Source: Nat Commun. 2021 Jul 5;12:4121. doi: 10.1038/s41467-021-24323-1 (PMC8257611; doi:10.1038/s41467-021-24323-1)
Supplement: Supplementary file 1 — Supplementary Information [file 41467_2021_24323_MOESM1_ESM.pdf]

SUPPLEMENTARY INFORMATION

Post-capillary venules are the key locus for transcytosis-mediated brain delivery of therapeutic nanoparticles

Authors: Krzysztof Kucharz[†1], Kasper Kristensen[†2], Kasper Bendix Johnsen[†2], Mette Aagaard Lund[2], Micael Lønstrup[1], Torben Moos[3], Thomas Lars Andresen[2], Martin Johannes Lauritzen[1,4].

Affiliations:

[1] Department of Neuroscience, Faculty of Health Sciences, University of Copenhagen, DK-2200, Copenhagen N, Denmark

[2] Department of Health Technology, Technical University of Denmark, DK-2800, Kongens Lyngby, Denmark

[3] Department of Health Science and Technology, Aalborg University, DK-9220, Aalborg Ø, Denmark

[4] Department of Clinical Neurophysiology, Rigshospitalet, DK-2600, Glostrup, Denmark

Footnotes:

Correspondence should be addressed to K.K. (e-mail: kucharz@sund.ku.dk) and M. J. L. (e-mail: mlauritz@sund.ku.dk)

† These authors contributed equally to this work.

CONTENTS:

Supplementary Figures	(Pages 2-6)
Supplementary Table	(Page 7)
Supplementary Methods	(Page 8-10)
Supplementary Note	(Page 11)
Supplementary References	(Pages 12-13)

SUPPLEMENTARY FIGURES

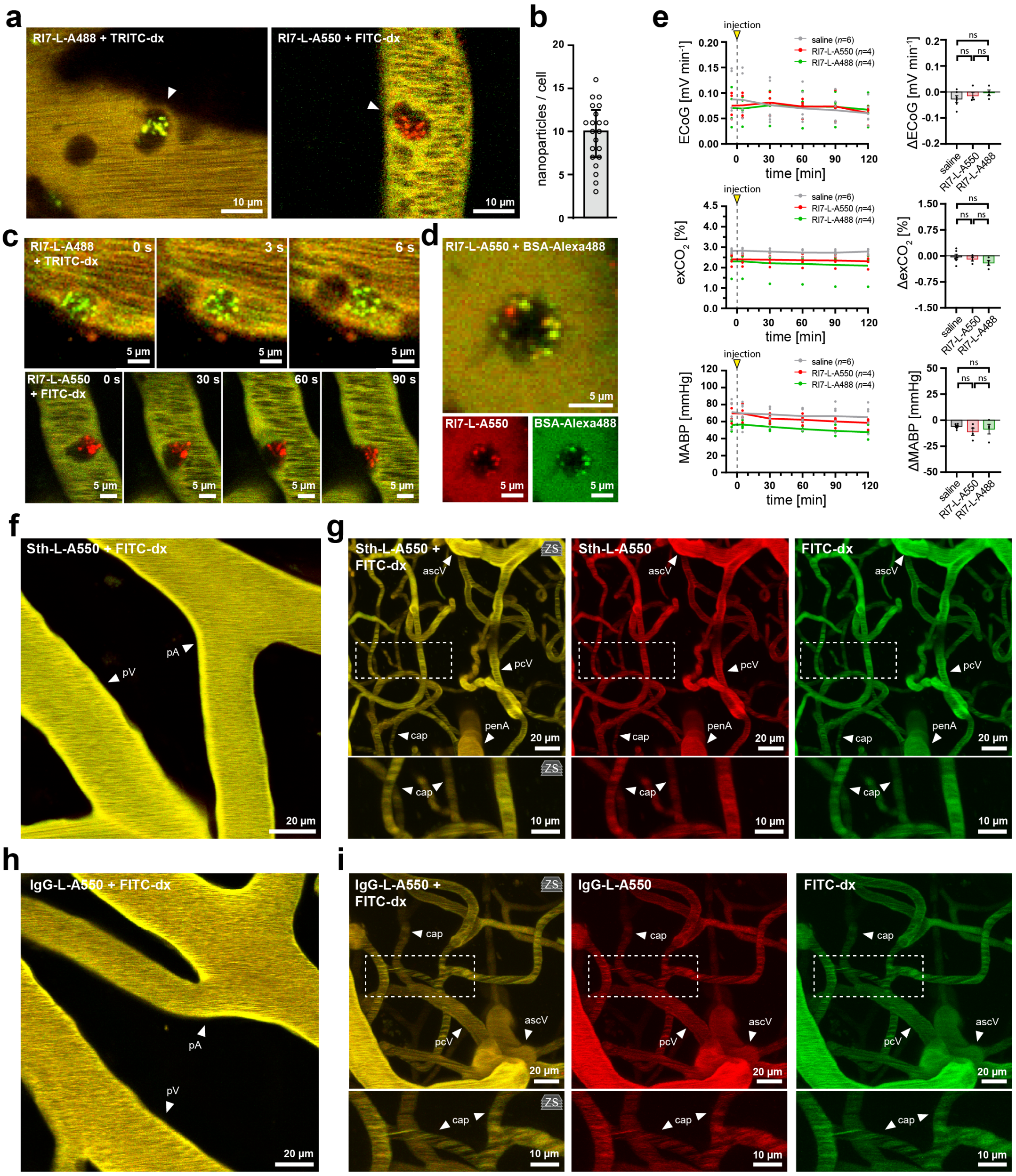

Supplementary Fig. 1. Lack of systemic adverse effects and unspecific binding of nanoparticles in the brain. a Blood-circulating leukocytes (arrowheads) with sequestered nanoparticles. See also Supplementary Movie S2. **b** On average, nanoparticle-positive leukocytes take up 10 nanoparticles. *n*=21 cells (in 5 mice). **c** Leukocytes with sequestered nanoparticles preserve their rolling and endothelium adherence properties. **d** The uptake of nanoparticles to leukocytes is unlikely driven by the RI7217 moiety, as it is also present, e.g., for fluorescently labeled albumin (BSA-Alexa488). **e** No significant effect of nanoparticles on brain activity (electrocorticogram, ECoG), exhaled CO_2 levels (exCO_2), and mean arterial blood pressure (MABP) at 2 h post-injection. *n*=4 mice (RI7-L-A550, RI7-L-488); *n=6* mice (saline control), two-tailed t-tests with Bonferroni correction, ns=not significant. **f,g** No association of stealth Atto 550-tagged nanoparticles (Sth-L-A550) to venules and capillaries. **h,i** No association of isotype IgG Atto 550-tagged nanoparticles (IgG-L-A550) to venules and capillaries. **All panels:** pV=pial venule; pcV=post-capillary venule; ascV=ascending venule; cap=capillaries; penA=penetrating arteriole; pA=pial arteriole. 'zs' inset indicates maximum intensity Z-stack projection. Data are means±SEM.

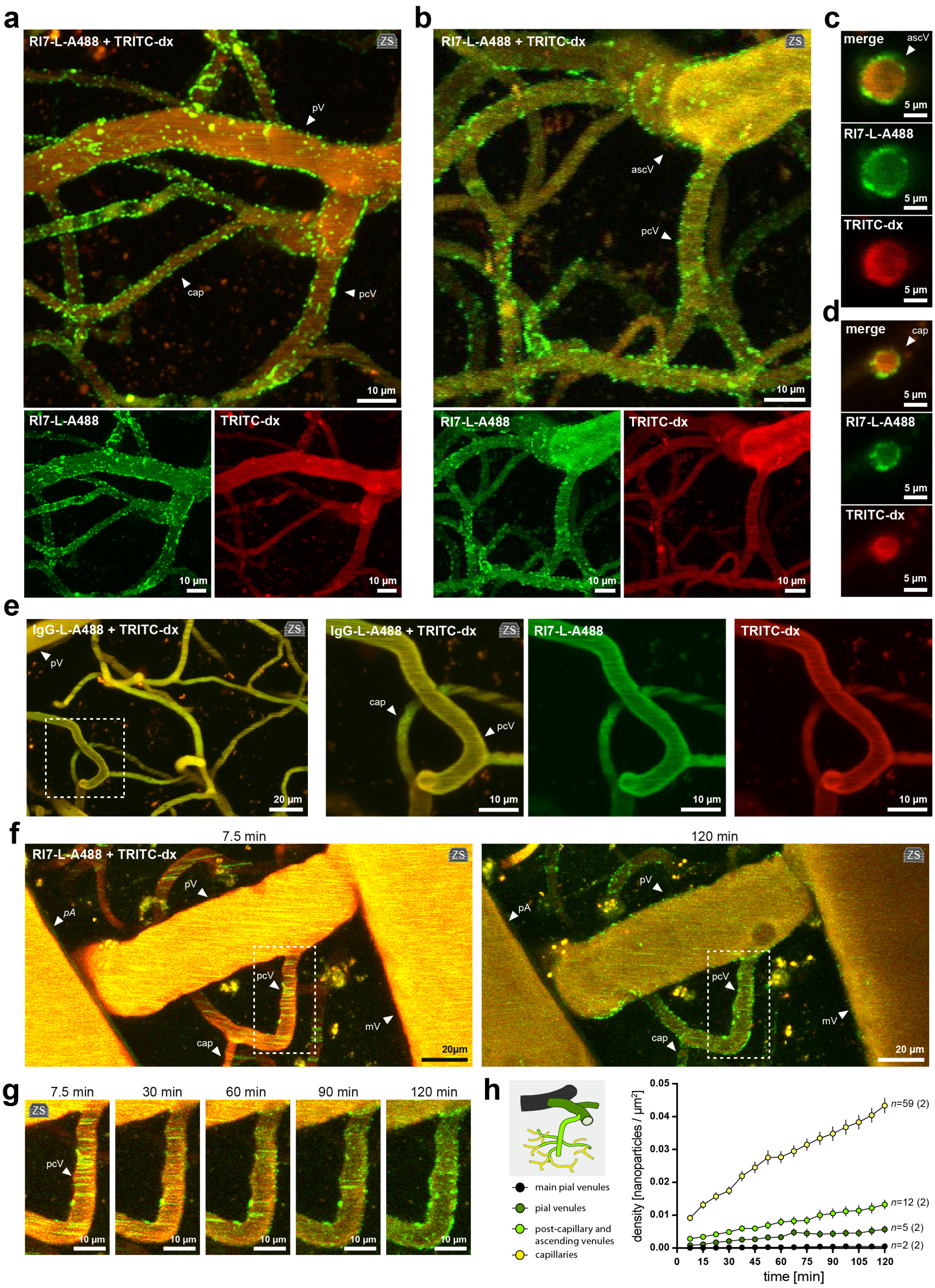

Supplementary Fig. 2. RI7-L-A488 follows RI7-L-A550 spatio-temporal distribution. a,b,c,d High resolution *in vivo* images of RI7-L-A488 nanoparticles (green) 2 h post-injection. Co-injected circulating TRITC-dx delineates vessel lumen (red). Nanoparticles are readily present at pial, ascending venules, post-capillary venules, and capillaries. **e** No association of isotype IgG A488-tagged nanoparticles to the brain microvasculature. **f,g,h** Association of RI7-L-A488 to vessel walls over time. The increase in associated nanoparticles over time is fastest at the capillaries and slowest at the large venules. Nanoparticle density = # of nanoparticles per µm^2 vessel wall area. Inset illustrates vessel hierarchy and color-coding. *n*=number of vessels, where n_{mV}=2*;* n_{pV}=5*;* $n_{\mathrm{pcV\_ascV}}$=12*;* n_{cap}=59 across 2 mice. Data are means±SEM (Supplementary note). **All panels**: mV=main pial venule; pV=pial venule; ascV=ascending venule; pcV=post-capillary venule; cap=capillaries; pA=pial arteriole. 'zs' inset indicates maximum intensity Z-stack projection.

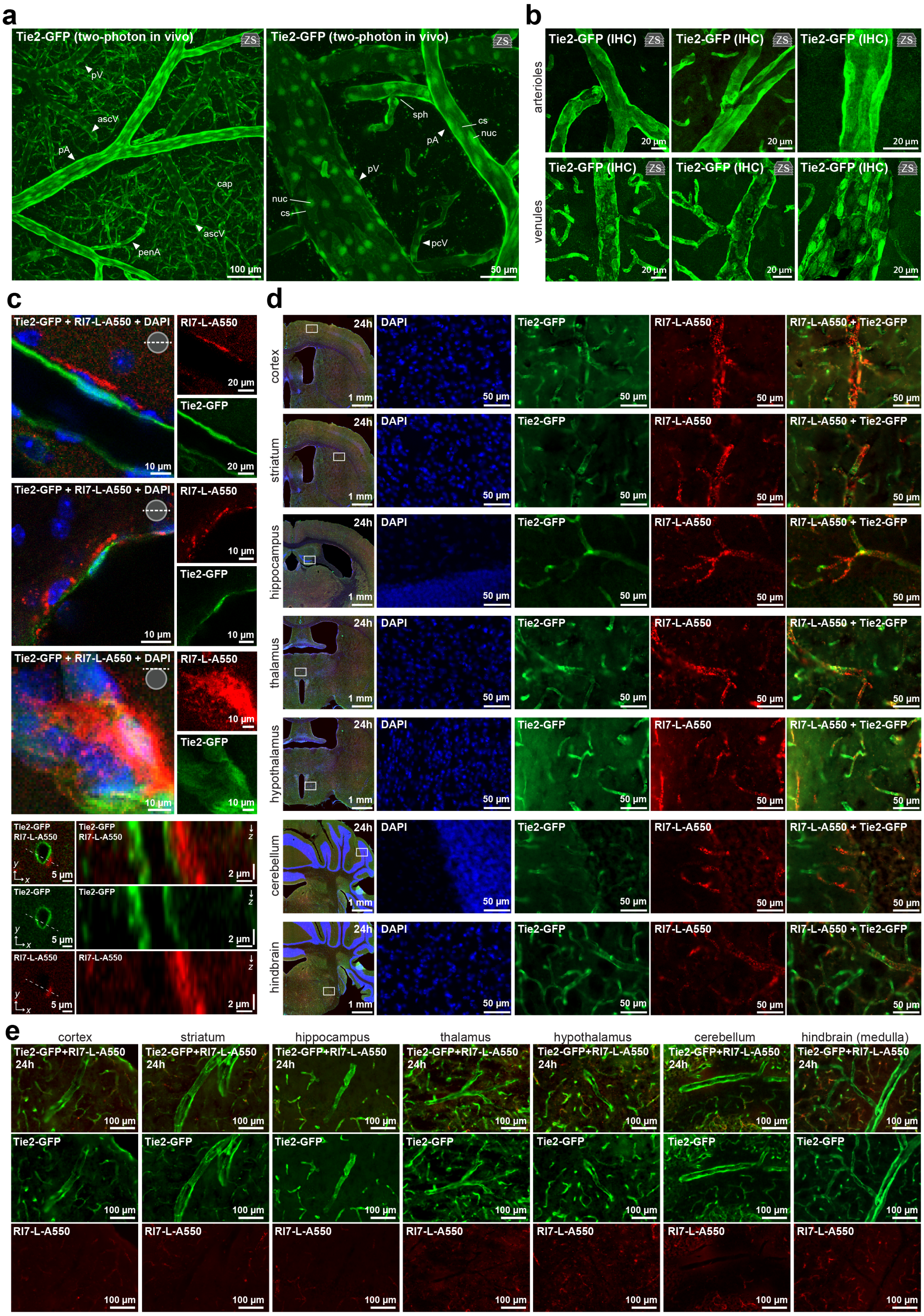

Supplementary Fig. 3. Immunohistochemistry preserves vessel morphology, but not nanoparticle integrity or perivascular spaces. a Z-stack projections of the brain cortical microvasculature imaged in Tie2-GFP mice using two-photon microscopy in vivo. **b** Following immunohistochemistry, the morphological features of arterioles and venules are generally preserved. **c** In contrast, immunohistochemistry perfusion-fixation collapses perivascular space around vessels affecting the distribution of nanoparticles on the abluminal side of the endothelium. In addition, it

compromises the nanoparticle structural integrity (demonstrated by the presence of a diffuse nanoparticle signal). Image insets denote the imaging plane in relation to a vessel perimeter. **d** The post-fixation artifacts effectively obscure the dissemination of nanoparticle location in relation to the endothelium, perivascular space, and the brain parenchyma regardless of the brain region. **e** In contrast to venules, no nanoparticles were detected in the vicinity of arterioles. **d,e** Images were collected from brains fixed for immunohistochemistry 24 hours after injection of RI7-L-A550. Tie2-GFP and RI7-L-A550 nanoparticles were quenched during tissue perfusion fixation, and were visualized by immunostaining (Supplementary Methods). **All panels:** IHC= immunohistochemistry; pV=pial venule; ascV=ascending venule; pcV=post-capillary venule; cap=capillaries; penA= penetrating arteriole; pA=pial arteriole; nuc=nucleus; cs=cell boundaries/endothelium contact sites; sph=precapillary sphincter. 'zs' inset indicates maximum intensity Z-stack projection.

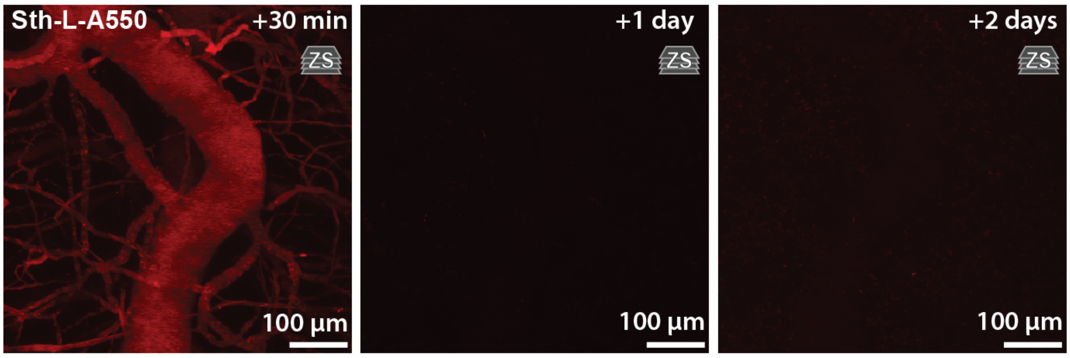

Supplementary Fig. 4. Stealth nanoparticles do not cross the BBB. a Long-term imaging of somatosensory cortex following the injection of Sth-L-A550. Lack of Sth-L-A550 presence in the brain, both at 24 and 48 h post-injection, attests for the absence of passive entry of nanoparticles to the brain and for the preserved BBB integrity during chronic imaging. All images are Z-stack maximum intensity projections from the same brain region. All images are presented in the same fluorescence intensity scale. Time is relative to Sth-L-A550 injection. 'zs' inset indicates maximum intensity Z-stack projection.

SUPPLEMENTARY TABLE

	Nanoparticle	D_h [nm]	PDI	Zeta potential [mV]	Fluorescence [arb. u.]	Antibody [g/mol lipid]
Fluorescent nanoparticles	Sth-L-A488	120	0.04	-8	17000	0
	Sth-L-A550	117±4	0.03±0.02	-7±1	13000±6000	0
	RI7-L-A488	139	0.13	-10	16000	29
	RI7-L-A550	136±4	0.12±0.01	-6±1	10000±3000	35±4
	IgG-L-A488	135	0.10	-9	14000	23
	IgG-L-A550	136±5	0.10±0.03	-7±1	9000±2000	31±20
	Nanoparticle	D_h [nm]	PDI	Cisplatin [g/mol lipid]		Antibody [g/mol lipid]
Cisplatin-loaded nanoparticles	Sth-L-Pt	118	0.03	15		0
	RI7-L-Pt	161	0.16	13		66
	IgG-L-Pt	168	0.13	13		89

Supplementary Table 1. Characteristics of nanoparticles. D_h=hydrodynamic diameter; PDI=polydispersity index; arb. u. = arbitrary unit. Data are means±SD.

SUPPLEMENTARY METHODS

Immunohistochemistry

The Tie2-GFP mice were perfused transcardially with ice-cold PBS for 1 min, then 4 % paraformaldehyde (PFA) solution in PBS for 7 min at the rate of 10 ml/min. The brains were carefully extracted and placed in a 4 % PFA for 24 h post-fixation at 4°C, then transferred to a 30 % sucrose solution in PBS and allowed to settle for 48 h at 4°C. Next, the brains were embedded in the Tissue-Tek O.C.T. compound and mounted onto a cryostat for sectioning. The brains were serially sectioned with a thickness of 40 μm resulting in six series of coronal sections covering the entire brain. The sections were kept in a cryopreserving solution at -20°C until the immunohistochemical (IHC) procedure.

IHC was performed to restore the signal intensity of the endogenous GFP signal in the transgenic mice and to visualize the antibodies conjugated to the liposome surface as a phantom of the lipid-fluorophore signal that is readily lost during the IHC procedure. The brain sections were washed 3x in PBS and blocked with a solution of 3 % swine serum and 0.3 % Triton X-100 in PBS for 2 h. A primary antibody solution was then added containing rabbit anti-GFP (1:500 dilution, ab6556 polyclonal; Abcam), and the brain sections were incubated overnight at 4°C on an orbital shaker. After incubation, the brain sections were washed 3x in PBS and incubated with a secondary antibody solution for 60 min containing goat anti-rabbit IgG with AlexaFluor488 (1:200 dilution, A11034, LifeTechnologies), and goat anti-rat IgG with AlexaFluor594 (1:200 dilution, A11007, LifeTechnologies). Next, the sections were washed 3x for 5 min in PBS, and incubated in a 2.8 nM DAPI solution followed by three additional washes. The sections were mounted onto glass slides and allowed to dry, and then were coverslipped with Fluorescence Mounting Medium (DAKO).

Overview of the brain sections were obtained via serial tile scanning using an Axio Scan.Z1 microscope (Carl Zeiss AG) with a Plan Apochromat 10 × objective (NA 0.45) and an F66-887 quadricolor filter (Semrock). Detailed images were obtained using a DMi8 microscope (Leica Microsystems) with a 40 × 1.3 NA oil immersion objective. The emitted light was collected after: 445–475 nm (for DAPI), 500–520 nm (for Alexa488–conjugated antibody), and 600–640 nm (for Alexa594–conjugated antibody) bandpass filter by a hybrid detector. The fluorophores were excited at 450 nm (DAPI), 488 nm (Alexa488-conjugated antibody), and 552 nm (Alexa594–conjugated antibody). The images were exported as 16-bit color depth tiff files to ImageJ (v. 1.52a; NIH) for further analysis.

Considerations of adverse systemic interactions

Liposome nanoparticles can be functionalized with antibodies using the post-insertion technique[1], or the post-functionalization approach[2, 3]. Post-insertion technique comes with the issue that lipid-conjugated antibodies that were not integrated into the liposomes may elicit adverse effects, typically attributed to retained effector function of the antibody Fc domain[4]. Animals exhibit rapid loss of locomotion accompanied with signs of limb spasticity within 5 min post-injection, and in 20% of cases, death[5]. Notably, this was not observed for the nanoparticles used herein, where the antibodies were post-functionalized to the liposome surface, consistent with our previous reports, showing lack of adverse effects for gold nanoparticles[6] or liposomes[7] formulated with the post-functionalization approach. In contrast to other TfR ligands, RI7217 does not compete with endogenous transferrin, and TfR vascular expression is highly specific to the brain[8]. Earlier studies using a similar system of TfR-targeted immunoliposomes reported no side effects, e.g., neuroinflammation, during chronic weekly administrations[9]. Here, the nanoparticles were coated with poly(ethylene glycol; PEG) to reduce nanoparticle recognition by the immune system[10, 11]. This may result in increased nanoparticle clearance due to the production of PEG-specific antibodies over subsequent injections, which was suggested to potentially change the pharmacokinetic profile, but without compromising immune system function[10]. In addition, we designed the nanoparticles to have near-neutral zeta potential (-10 to -7 mV, Supplementary Table 1). This is of advantage, as a high negative or positive charge of nanoparticles may promote the formation of protein corona that interferes with nanoparticle targeting[12]; and increase nanoparticle exposure to the immune system, facilitating their uptake by phagocytes[13, 14]. Lastly, nanoparticle formulations used herein do not also alter the TEER values in vitro, indicating a retained function of the paracellular barrier, as shown in our previous study[7]. Here, we observed no pathological changes in exhaled CO_2 levels, mean arterial blood pressure MABP, or brain activity after nanoparticle administration in acute experiments. In chronic experiments, we observed no signs of neuroinflammation, and the animals exhibited normal behavior (e.g., running, exploring, appetite) during and in between imaging sessions, with no observable

differences before (24 h prior) and after (30 min, 24 h, 48 h) administration of nanoparticles. In addition, no adverse effects were observed in animals treated with liposomes with encapsulated cisplatin in ICP-MS experiments.

Vessel classification criteria

Defining the reliable molecular markers to distinguish capillaries from the immediate downstream of blood flow post-capillary venules is complicated due to different transcriptome cluster classification criteria used in brain endothelium single-cell mRNA analyses, where the post-capillary segment is either not defined[15, 16] or a practical term of, e.g., "capillary-venous" segment is used, but without an anatomical foothold[17]. Here, following most two-photon imaging protocols, we traced vessel connectivity along the blood flow, and relied on vessel diameter and branching classification criteria. Two-photon imaging in vivo reveals that capillary lumen diameters range in mice on average from 10 to 5 μm[18, 19, 20, 21], from the ~10 μm capillaries that stem from pre-capillary arterioles to terminal (~6th) branching orders of capillaries which do not exceed 5 μm diameter[21]. Following that, microvessels converge, and vascular topology classifies coalesced vessels with a diameter ~6-7 μm in mice as the post-capillary venules[22, 23]. This division is based on the presence of loops in the capillary network, which are absent in post-capillary venules[22], and converges with functional assessments of the brain microvasculature, where ~7 μm microvessels are not considered to be a part of the capillary segment, but post-capillary venules[24]. This division is further reinforced by the distinct morphology of mural cells, where capillaries at the venular end are covered by pericytes with a large cell body and numerous branches, whereas functionally and morphologically distinct 'mesh-like' (a.k.a. stellate or post-capillary) pericytes reside at post-capillary venules[25, 26]. The post-capillary mesh-like pericytes occupy vessels with diameters larger than ~6-7 μm[26, 27]. In addition, the lack of perivascular space at capillaries further distinguishes them from post-capillary venules. The perivascular space is absent for vessels with a diameter of 6 μm or less, in contrast to the presence of perivascular space in post-capillary venules, at microvessels with a diameter larger than 6 μm[28].

Here, using the morphological and functional classification criteria above, we considered post-capillary venules as microvessels with a diameter larger than 6 μm and coalescing from two parent vessels located upstream of the blood flow.

SUPPLEMENTARY NOTE

For clarity, the data from Fig. 2e (RI7-L-A550) and Supplementary Fig. 2h (RI7-L-A488) showing changes in nanoparticle density over time were represented as means±SEM. For samples *n*<10, we also present the data from Fig. 3e and Supplementary Fig. 2h with individual values, i.e., each point corresponding to a single vessel in a respective time-point. Lines are averages, *n*= number of vessels analyzed across a number of mice (in brackets).

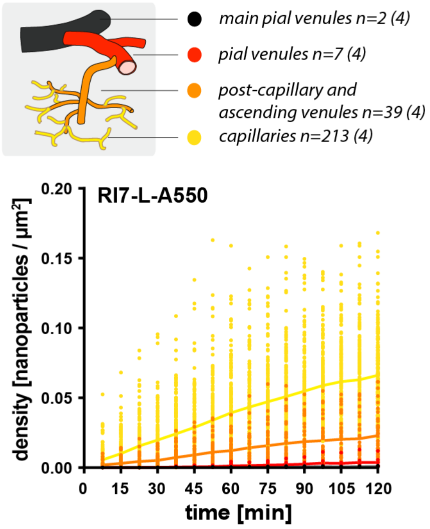

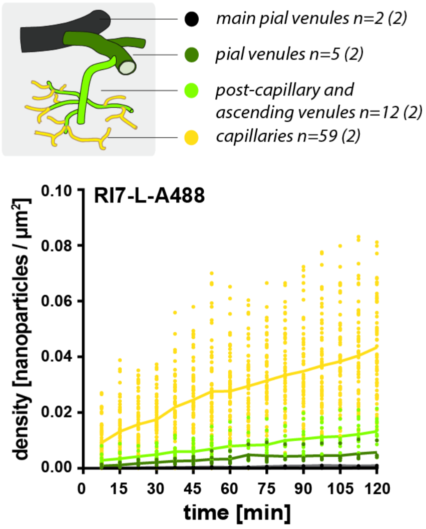

SUPPLEMENTARY REFERENCES

1. Moreira JN, Ishida T, Gaspar R, Allen TM. Use of the post-insertion technique to insert peptide ligands into pre-formed stealth liposomes with retention of binding activity and cytotoxicity. *Pharm Res* **19**, 265-269 (2002).

2. Kristensen K, Engel TB, Stensballe A, Simonsen JB, Andresen TL. The hard protein corona of stealth liposomes is sparse. *J Control Release* **307**, 1-15 (2019).

3. Bak M, Jolck RI, Eliasen R, Andresen TL. Affinity Induced Surface Functionalization of Liposomes Using Cu-Free Click Chemistry. *Bioconjug Chem* **27**, 1673-1680 (2016).

4. Couch JA*, et al.* Addressing safety liabilities of TfR bispecific antibodies that cross the blood-brain barrier. *Sci Transl Med* **5**, 183ra157, 181-112 (2013).

5. Johnsen KB*, et al.* Modulating the antibody density changes the uptake and transport at the blood-brain barrier of both transferrin receptor-targeted gold nanoparticles and liposomal cargo. *J Control Release* **295**, 237-249 (2019).

6. Johnsen KB*, et al.* Antibody affinity and valency impact brain uptake of transferrin receptor-targeted gold nanoparticles. *Theranostics* **8**, 3416-3436 (2018).

7. Johnsen KB*, et al.* Targeting transferrin receptors at the blood-brain barrier improves the uptake of immunoliposomes and subsequent cargo transport into the brain parenchyma. *Sci Rep* **7**, 10396 (2017).

8. van Rooy I, Mastrobattista E, Storm G, Hennink WE, Schiffelers RM. Comparison of five different targeting ligands to enhance accumulation of liposomes into the brain. *J Control Release* **150**, 30-36 (2011).

9. Zhang YF, Boado RJ, Pardridge WM. Absence of toxicity of chronic weekly intravenous gene therapy with pegylated immunoliposomes. *Pharm Res* **20**, 1779-1785 (2003).

10. Dobrovolskaia MA, McNeil SE. Immunological properties of engineered nanomaterials. *Nat Nanotechnol* **2**, 469-478 (2007).

11. Moghimi SM. Chemical camouflage of nanospheres with a poorly reactive surface: towards development of stealth and target-specific nanocarriers. *Biochimica et biophysica acta* **1590**, 131-139 (2002).

12. Lundqvist M, Stigler J, Elia G, Lynch I, Cedervall T, Dawson KA. Nanoparticle size and surface properties determine the protein corona with possible implications for biological impacts. *Proc Natl Acad Sci U S A* **105**, 14265-14270 (2008).

13. Xiao K*, et al.* The effect of surface charge on in vivo biodistribution of PEG-oligocholic acid based micellar nanoparticles. *Biomaterials* **32**, 3435-3446 (2011).

14. Nakanishi T*, et al.* Positively charged liposome functions as an efficient immunoadjuvant in inducing cell-mediated immune response to soluble proteins. *J Control Release* **61**, 233-240 (1999).

15. Sabbagh MF*, et al.* Transcriptional and epigenomic landscapes of CNS and non-CNS vascular endothelial cells. *Elife* **7**, (2018).

16. Vanlandewijck M*, et al.* A molecular atlas of cell types and zonation in the brain vasculature. *Nature* **554**, 475-480 (2018).

17. Kalucka J*, et al.* Single-Cell Transcriptome Atlas of Murine Endothelial Cells. *Cell* **180**, 764-779 e720 (2020).

18. Cai C*, et al.* Stimulation-induced increases in cerebral blood flow and local capillary vasoconstriction depend on conducted vascular responses. *Proc Natl Acad Sci U S A* **115**, E5796-E5804 (2018).

19. Khennouf L*, et al.* Active role of capillary pericytes during stimulation-induced activity and spreading depolarization. *Brain* **141**, 2032-2046 (2018).

20. Kutuzov N, Flyvbjerg H, Lauritzen M. Contributions of the glycocalyx, endothelium, and extravascular compartment to the blood-brain barrier. *Proc Natl Acad Sci U S A* **115**, E9429-E9438 (2018).

21. Hartmann DA*, et al.* Brain capillary pericytes exert a substantial but slow influence on blood flow. *Nat Neurosci*, (2021).

22. Cruz Hernandez JC*, et al.* Neutrophil adhesion in brain capillaries reduces cortical blood flow and impairs memory function in Alzheimer's disease mouse models. *Nat Neurosci* **22**, 413-420 (2019).

23. Smith AF*, et al.* Brain Capillary Networks Across Species: A few Simple Organizational Requirements Are Sufficient to Reproduce Both Structure and Function. *Front Physiol* **10**, 233 (2019).

24. Sakadzic S*, et al.* Large arteriolar component of oxygen delivery implies a safe margin of oxygen supply to cerebral tissue. *Nat Commun* **5**, 5734 (2014).

25. Uemura MT, Maki T, Ihara M, Lee VMY, Trojanowski JQ. Brain Microvascular Pericytes in Vascular Cognitive Impairment and Dementia. *Front Aging Neurosci* **12**, 80 (2020).

26. Hartmann DA, Underly RG, Grant RI, Watson AN, Lindner V, Shih AY. Pericyte structure and distribution in the cerebral cortex revealed by high-resolution imaging of transgenic mice. *Neurophotonics* **2**, 041402 (2015).

27. Berthiaume AA, Hartmann DA, Majesky MW, Bhat NR, Shih AY. Pericyte Structural Remodeling in Cerebrovascular Health and Homeostasis. *Front Aging Neurosci* **10**, 210 (2018).

28. Owens T, Bechmann I, Engelhardt B. Perivascular spaces and the two steps to neuroinflammation. *J Neuropathol Exp Neurol* **67**, 1113-1121 (2008).
