# Supplementary material for: Post-capillary venules are the key locus for transcytosis-mediated brain delivery of therapeutic nanoparticles
Source: Nat Commun. 2021 Jul 5;12:4121. doi: 10.1038/s41467-021-24323-1 (PMC8257611; doi:10.1038/s41467-021-24323-1)
Supplement: Supplementary file 3 — Description of Additional Supplementary Files [file 41467_2021_24323_MOESM3_ESM.docx]

**Description of Additional Supplementary Files**

**Supplementary Movie 1.** Blood-circulating nanoparticles show high fluorescence stability over time. Time is relative to the time of intravenous nanoparticle injection.

**Supplementary Movie 2.** A small fraction of nanoparticles is sequestered by circulating leukocytes.

**Supplementary Movie 3.** Nanoparticle association to the brain microvasculature over time. Concurrent imaging of RI7-L-A550 nanoparticles (red) with FITC-dextran (FITC-dx, green). Time is relative to the time of nanoparticle injection.

**Supplementary Movie 4.** Time-lapse recording of nanoparticle (RI7-L-A550) movement in the brain endothelial cells. The endothelium is visible in green (Tie2-GFP). Left panels: pial venule; right panel: post-capillary venule.

**Supplementary Movie 5.** Time-lapse recording of nanoparticle (RI7-L-A550) motility at the capillary segment with examples of nanoparticle tracing. Circles denote example nanoparticles; crosses correspond to nanoparticle positions.

**Supplementary Movie 6.** Nanoparticles exhibit movement at vessel walls, even in stalled capillaries.

**Supplementary Movie 7.** Right panel: nanoparticle (RI7-L-A550, red) transcytosis into the perivascular space at the level of post-capillary venules. Left panel: no apparent transcytosis in capillaries. The vessel lumen is delineated by circulating FITC-dextran (FITC-dx, green). The white circle denotes transcytosed nanoparticle, the cyan circle is a subsequent nanoparticle entering a similar route.

**Supplementary Movie 8.** Time-lapse recording of nanoparticle (RI7-L-A550, red) progressing in the brain parenchyma. The endothelium is visible in green (Tie2-GFP). The white circle denotes moving nanoparticle.

**Supplementary Movie 9.** Nanoparticles after laser extravasation from the blood to the brain. No significant nanoparticle movement in proximity to capillaries. In contrast, rapid nanoparticle progression in perivascular space in proximity to venules. Lines denote vessel lumen boundaries.

**Supplementary Movie 10.** Leukocyte entry into the brain with previously sequestered nanoparticles (RI7-L-A550, red) from the blood circulation. Vessel lumen is delineated by circulating FITC-dextran (FITC-dx, green).
