# Supplementary material for: Post-capillary venules are the key locus for transcytosis-mediated brain delivery of therapeutic nanoparticles
Source: Nat Commun. 2021 Jul 5;12:4121. doi: 10.1038/s41467-021-24323-1 (PMC8257611; doi:10.1038/s41467-021-24323-1)
Supplement: Supplementary file 14 — Reporting Summary [file 41467_2021_24323_MOESM14_ESM.pdf]

nature research

Corresponding author(s): Krzysztof Kucharz

Last updated by author(s): Jun 1, 2021

Reporting Summary

Nature Research wishes to improve the reproducibility of the work that we publish. This form provides structure for consistency and transparency in reporting. For further information on Nature Research policies, see our Editorial Policies and the Editorial Policy Checklist.

Statistics

For all statistical analyses, confirm that the following items are present in the figure legend, table legend, main text, or Methods section.

n/a	Confirmed	
☐	☒	The exact sample size (n) for each experimental group/condition, given as a discrete number and unit of measurement
☐	☒	A statement on whether measurements were taken from distinct samples or whether the same sample was measured repeatedly
☐	☒	The statistical test(s) used AND whether they are one- or two-sided<br>*Only common tests should be described solely by name; describe more complex techniques in the Methods section.*
☒	☐	A description of all covariates tested
☐	☒	A description of any assumptions or corrections, such as tests of normality and adjustment for multiple comparisons
☐	☒	A full description of the statistical parameters including central tendency (e.g. means) or other basic estimates (e.g. regression coefficient) AND variation (e.g. standard deviation) or associated estimates of uncertainty (e.g. confidence intervals)
☐	☒	For null hypothesis testing, the test statistic (e.g. F, t, r) with confidence intervals, effect sizes, degrees of freedom and P value noted<br>*Give P values as exact values whenever suitable.*
☒	☐	For Bayesian analysis, information on the choice of priors and Markov chain Monte Carlo settings
☒	☐	For hierarchical and complex designs, identification of the appropriate level for tests and full reporting of outcomes
☒	☐	Estimates of effect sizes (e.g. Cohen's d, Pearson's r), indicating how they were calculated

Our web collection on statistics for biologists contains articles on many of the points above.

Software and code

Policy information about availability of computer code

Data collection	LAS AF (v. 4.4 in vivo; Leica Microsystems); LAS X (v. 3.5.7 ex vivo; Leica Microsystems); ZEN Slidescan (v. 2012; Zeiss); Spike2 (v. 7.02a; CED)
Data analysis	ImageJ (v. 1.52a; NIH); Amira v. 6 (FEI Visualization Sciences Group); Prism v.8.2 (GraphPad); OriginPro 2018 (OriginLab Corporation)

For manuscripts utilizing custom algorithms or software that are central to the research but not yet described in published literature, software must be made available to editors and reviewers. We strongly encourage code deposition in a community repository (e.g. GitHub). See the Nature Research guidelines for submitting code & software for further information.

Data

Policy information about availability of data

All manuscripts must include a data availability statement. This statement should provide the following information, where applicable:

- Accession codes, unique identifiers, or web links for publicly available datasets
- A list of figures that have associated raw data
- A description of any restrictions on data availability

The data that support the findings of this study are available from the corresponding author upon reasonable request. The source data underlying Figs. 1d, 1g, 1j, 3c, 3e, 4f, 5d, 5e, 5f, 5k, 6e, 6j, Supplementary Fig. 1e and Supplementary Fig. 2h are provided as a Source Data file.

Field-specific reporting

Life sciences study design

All studies must disclose on these points even when the disclosure is negative.

Sample size	This study was observational and explored how different vessel segments handled individual nanoparticles, and not based on comparisons between animal groups. The sample size was not predetermined before the study due to the lack of existing estimates in the literature. To quantitatively characterize the average magnitude of nanoparticle properties in the brain, the statistical parameters were the number of mice (in Fig. 1d n=4, 4, 5; Supplementary Fig. 1e n=4, 4, 6); distinct vessel segments (in Fig. 3c,e n_vessels_total = 261 in 4 mice; Supplementary Fig. 2h = 78 in 2 mice); or number of nanoparticles to characterize their average size (in Fig. 1j n= 20 nanoparticles per each type of a nanoparticle across 2 mice); location in relation to vascular endothelium (in Fig. 4 f,g n_nanoparticles=1068 in 5 mice); subcellular distribution in the endothelial cells (Fig.5 k n_nanoparticles=3534 in 5 mice); or movement dynamics (Fig. 5c,d,e,f n_total_nanoparticles=670 across 67 vessels in 5 mice). In Fig. 6d,e, we used 2 animals to demonstrate the robust differences in the movement of extravasated nanoparticles at different vessel types (n_nanoparticles=64 per each analyzed vessel type, imaged during 2 separate extravasations, from 2 different brain areas per mouse). To demonstrate binary result (i.e., the presence vs. absence of nanoparticles in the brain) in Fig. 6j we used 2 animals in vivo, and independently, in Supplementary Fig. 3c,d 2 animals ex vivo. Upon confirmation, nanoparticle distribution was quantified using n= 1256 nanoparticles imaged across 2 distinct brain areas per mouse. The sample sizes were sufficient to determine the nanoparticle location in the brain, movement dynamics, and the ability of nanoparticles to cross the blood-brain barrier.
Data exclusions	Exclusions concerning animal welfare were pre-established based on our previous experience with two-photon imaging in vivo (e.g. PMID: 24670647; PMID: 29538620). Prior to injection of nanoparticles, all animals with abnormal blood pressure (<50 mmHg), abnormal brain ECoG activity, or significant (>2 µm/min) brain movement in x, y, or z coordinates were excluded from the study (3 excluded out of total 36 animals prepared for in vivo imaging). No animals were excluded from immunohistochemistry analysis (total = 2 mice injected with RI7-L-A550 for confocal imaging) and from ICP-MS (total = 13 mice). A small percentage of nanoparticles, which exhibited diffuse signals that suggested nanoparticle destruction, or nanoparticles at the vessels with abnormal morphology, were excluded from the analysis.
Replication	We observed consistent replication of nanoparticle properties between biologically independent mice, with no presence of animal outliers. The numbers of replicates used to quantitatively characterize the average magnitude of observed effects were provided in the sample size section (above), in the figure legends and figures, and were sufficient to determine the nanoparticle location in the brain, movement dynamics, and the ability of nanoparticles to cross the blood-brain barrier. The representative images show scans from across n = mice with similar results, where: Fig. 1f n_RI7-L-A550=4, n_RI7-L-A488=4; Fig. 1h n_RI7-L-A550=3, n_RI7-L-A488=3; Fig. 1i n=1; Fig. 2a-f n=7; Fig. 2g-h n=1; Fig. 3a-b n=4; Fig. 3d n =4; Fig. 4a-e n=5; Fig. 5a-b n=5; Fig. 5g-i, l n=5; Fig. 6a n_upper_panel=4, n_lower_panel=3; Fig. 6b n=3 (n=1 for real-time recording capturing the whole event of transcytosis with addition of n =2 for partially captured process); Fig. 6c n=2; Fig. 6d n=4; Fig. 6g, h n=2; Supplementary Figs. 1a, c n_RI7-L-A550=6, n_RI7-L-A488=5; Supplementary Fig. 1d n=2; Supplementary Fig. 2a-d n=2; Supplementary Fig. 3a n=5. Supplementary Fig. 3b-e n=2. Images from single experiments have been provided to illustrate the lack of targeting properties of nanoparticles without TfR-targeting antibody, i.e., in Supplementary Fig. 1f-g n=1; Supplementary Fig. 1h-i n=1; Supplementary Fig. 2e n=1; Supplementary Fig. 4. n=1; which was in accord with results from Fig. 1d, and an expected nanoparticle feature found across the literature.
Randomization	The sample randomization was not performed. Except for experiments presented in Fig.1d,g, and Supplementary Fig. 1e, the randomization did not apply to the study, as we neither compared the properties of distinct nanoparticle formulations nor distinct animal groups, but investigated interactions of a single nanoparticle type at distinct vessel types.
Blinding	The blinding to experimental conditions was not performed. It was not possible to perform blinding during data collection in experiments presented in Fig.1g (nanoparticles and fluorescent dyes in circulation) and Fig. 1h (nanoparticles with two distinct fluorophores) due to different imaging parameters of imaged fluorophores. For all the remaining imaging datasets in the study, the blinding did not apply, as we did not compare the properties of distinct nanoparticle formulations or animal groups. There was no subjective assessment of nanoparticle properties (location in the brain, movement dynamics, and the ability of nanoparticles to cross the blood-brain barrier), as the outcomes were independent of the investigator's judgment.

Reporting for specific materials, systems and methods

We require information from authors about some types of materials, experimental systems and methods used in many studies. Here, indicate whether each material, system or method listed is relevant to your study. If you are not sure if a list item applies to your research, read the appropriate section before selecting a response.

Materials & experimental systems

n/a	Involved in the study	
☐	☒	Antibodies
☒	☐	Eukaryotic cell lines
☒	☐	Palaeontology and archaeology
☐	☒	Animals and other organisms
☒	☐	Human research participants
☒	☐	Clinical data
☒	☐	Dual use research of concern

Methods

n/a	Involved in the study	
☒	☐	ChIP-seq
☒	☐	Flow cytometry
☒	☐	MRI-based neuroimaging

Antibodies

Antibodies used

PRIMARY ANTIBODIES:
1) Rat anti-TfR antibody clone RI7217 to functionalize the nanoparticles. The antibody was produced in-house using the hybridoma technique at Laboratory for Neurobiology, Aalborg University, Denmark. The antibody specificity was previously determined using surface plasmon resonance [Johnsen KB, et al. Theranostics 8, 3416-3436 (2018)]. The application of nanoparticle-antibody conjugate has been the major topic investigated in this study.

2) IgG antibody Thermo Fisher Scientific (#02-9602)

3) rabbit anti-GFP (1:500 dilution, #ab6556 polyclonal; Abcam)

SECONDARY ANTIBODIES
4) anti-rabbit IgG with AlexaFluor488 (1:200 dilution, #A11034, LifeTechnologies)

5) anti-rat IgG with AlexaFluor594 (1:200 dilution, #A11007, LifeTechnologies)

Validation

DESCRIPTION OF PRIMARY ANTIBODIES

1) Antibody: RI7217, monoclonal
Target: Mouse transferrin receptor protein 1.
Source: Laboratory for Neurobiology, Aalborg, Denmark (produced from hybridoma and validated using surface plasmon resonance)
Host/Isotype: Rat IgG2a
Reactivity: Mouse
Applications: IHC; ELISA
Citation#1: Johnsen KB, et al. Antibody affinity and valency impact brain uptake of transferrin receptor-targeted gold nanoparticles. Theranostics 8, 3416-3436 (2018).
Citation#2: Lee HJ, Engelhardt B, Lesley J, Bickel U, Pardridge WM. Targeting rat anti-mouse transferrin receptor monoclonal antibodies through blood-brain barrier in mouse. J Pharmacol Exp Ther 292, 1048-1052 (2000).
RRID: N/A
In this study: used to target nanoparticles to the brain endothelium to enable nanoparticle transcytosis (nanoparticle formulations: RI7-L-A550; RI7-L-A488).
Link: N/A

2) Antibody: Rat IgG Isotype Control
Target: Gamma immunoglobulin
Source: Thermo Fisher Scientific (#02-9602)
Host/Isotype: Rat IgG2a isotype control
Reactivity: N/A
Applications: Western Blot; IHC; ELISA; flow cytometry
Citations: N/A
RRID: AB_2532969
In this study: used as a negative control for nanoparticle targeting to the transferrin receptor (nanoparticle formulation: IgG-L-A550).
Link: https://www.thermofisher.com/antibody/product/Rat-IgG-Isotype-Control/02-9602

3) Antibody: Anti-GFP
Target: Green Fluorescent Protein
Source: Abcam (#6556)
Host/Isotype: Rabbit IgG
Reactivity: Aequorea victoria GFP such as S65T-GFP, RS-GFP, YFP, CFP, RFP and EGFP.
Applications: TEM, ICC
Citation #1: Hoermann B et al. Dissecting the sequence determinants for dephosphorylation by the catalytic subunits of phosphatases PP1 and PP2A. Nat Commun 11:3583 (2020).
Citation #2: Bojarskaite L et al. Astrocytic Ca2+ signaling is reduced during sleep and is involved in the regulation of slow wave sleep. Nat Commun 11:3240 (2020).
RRID: AB_305564
In this study: used to restore endogenous GFP signal in the vascular endothelium after GFP quenching due to perfusion-fixation.
Link: https://www.abcam.com/gfp-antibody-ab6556.html

Animals and other organisms

Policy information about studies involving animals; ARRIVE guidelines recommended for reporting animal research

Laboratory animals	We used wild-type C57Bl/6 mice, age 5–7 months (23–31 g) and age-matched (25–32 g) homozygous Tg(TIE2GFP)287Sato/J transgenic reporter mice (Tie2-GFP mice, #003658, The Jackson Laboratory). All animals were housed in ventilated cages under a 12 h light/12 h dark cycle, at 50 +/- 10% relative humidity, at room temperature; with ad libitum access to food and water. The animal housing facility is accredited by the Association for Assessment and Accreditation of Laboratory Animal Care (AAALAC), and the Federation of Laboratory Animal Science Associations (FELASA).
Wild animals	This study did not involve wild animals
Field-collected samples	This study did not involve field-collected samples
Ethics oversight	All animal experiments were approved by the Danish National Committee on Health Research Ethics and followed ARRIVE guidelines

Note that full information on the approval of the study protocol must also be provided in the manuscript.
